# The feasibility and effectiveness of one-puncture of rectus sheath block combined with transverse abdominis plane block in patients undergoing thoracoscopic-laparoscopic radical esophagectomy: a prospective randomized controlled study

**DOI:** 10.3389/fmed.2025.1568464

**Published:** 2025-04-07

**Authors:** Jing Lin, Jinghao Yang, Yihang He, Xinghao Wang, Luoting Li, Youbo Zuo

**Affiliations:** Department of Anesthesiology, Affiliated Hospital of North Sichuan Medical College, Sichuan, China

**Keywords:** rectus sheath block, transverse abdominis plane block, esophagectomy, thoracoscopic–laparoscopic, nerve block

## Abstract

**Background:**

This study aimed to assess the feasibility and effectiveness of ultrasound-guided one-puncture of rectus sheath block (RSB) combined with the transverse abdominis plane block (TAPB) for patients undergoing thoracoscopic-laparoscopic radical esophagectomy (TLE).

**Methods:**

This prospective randomized controlled study enrolled 40 patients aged from 50 to 80 years who were eligible for TLE. The patients were randomly assigned into two groups: intervention group (one-puncture of RSB combined with TAPB) and control group (traditional RSB and TAPB). The primary outcome was the duration of the nerve block procedure, and the secondary endpoints in this study included the convenience of the nerve block operation, sufentanil consumption, visual analog scale (VAS) scores at 2, 4, 6, 12, 24 and 48 h after surgery, the Riker Sedation-Agitation Scale (SAS) score, postoperative nausea and vomiting (PONV), the first time of the need for rescue analgesic, time of first PCIA presses, the duration of the postoperative hospital stay, and the incidence of adverse reactions after surgery.

**Results:**

The duration of the nerve block procedure in the intervention group was significantly shorter than that in the control group (182.7 ± 13.9 s vs. 199.4 ± 10.9 s, *p* = 0.0003), and the convenience of the nerve block operation in the intervention group was significantly higher than that in the control group (*p* < 0.001). There were no statistically significant differences between the groups in terms of the RSAS score, VAS scores, total analgesic consumption, additional analgesic use, and adverse effects (*p* > 0.05).

**Conclusion:**

The one-puncture of RSB combined with TAPB could provide sufficient analgesia for patients undergoing TLE, and reduce the duration of the nerve block procedure and enhance the convenience of the nerve block operation compared to the traditional RSB and TAPB.

## Introduction

At present, the incidence of esophageal cancer remains fairly high in China (1, 2). Esophagectomy serves as the main treatment modality for resectable esophageal cancer, however, it significantly interferes with physiological functions and has a relatively high rate of postoperative complications (3). Open resection of esophageal cancer and Thoracoscopic-laparoscopic esophagectomy (TLE) are the common techniques employed for conducting esophagectomy, and TLE undoubtedly has significantly reduced the trauma for patients (4, 5). Nevertheless, this procedure involves three surgical areas, namely the left neck, abdomen, and upper right chest, resulting in an extensive surgical range (6). Hence, postoperative pain remains severe, which seriously affects the patients’ respiratory function exercise and expectoration of phlegm, ultimately leading to lung atelectasis, pulmonary infections, and other pulmonary complications that hamper postoperative recovery and prolong the hospitalization time. Alleviating the surgical stress response and managing postoperative pain are crucial components after TLE.

There are numerous analgesic techniques utilized for pain management during TLE, including epidural anesthesia, thoracic paravertebral nerve block, serratus anterior plane block, erector spinal plane block, and regional anesthesia (7–9). These analgesic techniques indeed offer a large pain-blocking area in the right chest, however, they are unable to relieve the pain from the abdomen and are also associated with certain risks such as infection, bleeding, and nerve damage (10). Currently, the transversus abdominis plane block (TAPB) is utilized for postoperative analgesia control following abdominal surgery (11–13). However, these studies also found that TAPB could not completely cover the pain from the abdominal median incision below the xiphoid, and the pain of the abdominal median incision below the xiphoid was severe, which caused some potential complications such as retained sputum and respiratory depression (14). Donohoe et al. found that rectus sheath catheters were effective for esophagectomy analgesia (15), and Li et al. indicated that rectus sheath block could provide better analgesia and more hemodynamic stability for gastrectomy (16). Thus someone used ultrasound-guided RSB combined with TAPB for analgesia (17).

RSB combined with TAPB indeed reduces postoperative opioid use in patients following laparoscopy-assisted surgery, but the duration of the nerve block procedure is long and the location procedures are complex, which can aggravate the patient’s discomfort, and multipoint repeated puncture increases the risk of infection. Therefore, we propose that one-puncture of RSB combines with TAPB for pain relief in TLE. Our previous study found that one-puncture of RSB combined with TAPB was feasible and could effectively reduce postoperative pain and promote recovery in laparoscopic upper abdominal surgery (18).

There have been no previous reports on the application of one-puncture of RSB combined with TAPB in thoracic surgery. Hence, this prospective study aims to explore the feasibility and effectiveness of ultrasound-guided one-puncture of RSB combined with TAPB in enhancing postoperative analgesia and postoperative recovery after TLE.

## Methods

### Patients

In this single-center prospective randomized controlled study focusing on TLE, the study was approved by the ethics committee of the Affiliated Hospital of North Sichuan Medical College (approval number 2022ER370-1).

A total of 49 patients undergoing TLE from December 2022 to December 2023 at the Affiliated Hospital of North Sichuan Medical College were enrolled. Each patient provided written informed consent prior to participating in the study-related procedures. The inclusion criteria were as follows: ASA I–III patients aged 50–80 years, BMI 18.5–30 kg/m^2^, scheduled for elective TLE. The exclusion criteria included: refusal of nerve block or the procedure, allergy to local anesthetics, infection at the puncture site, coagulopathy (INR >1.5, platelets <50 × 10^9^/L), or prior thoracic/abdominal surgery. In cases where nerve block application was unsuccessful (such as when the block needle could not reach the appropriate sites or the local anesthetic distribution was not satisfactory), conversion to thoracotomy or reoperation, blood loss exceeding 600 mL, or admission to the intensive care unit (ICU) after surgery, these patients were planned to be excluded. All patients were informed about the use of the patient controlled intravenous analgesia (PCIA) device and the visual analog scale (VAS) on the day before surgery.

All patients were allocated to the interventional group and the control group in a 1:1 ratio through a computer-generated randomization table. The randomized results were stored in a sealed envelope, and the staff responsible for data collection and analysis were unaware of the group allocations. The patients’ record charts were saved in another envelope until the completion of the trial.

### Anesthesia procedure

Regarding the anesthesia procedure, after the patients arrived at the anesthesia preparation room, venous access was routinely established, and they received standard monitoring, which included electrocardiography (ECG), oxygen saturation (SpO2), non-invasive blood pressure, and bispectral index monitoring (BIS). Additionally, invasive arterial pressure was monitored by inserting a radial artery cannula under local anesthesia.

#### Block procedures

All patients underwent US-guided nerve block under sedation with midazolam at a dosage of 1 mg and analgesia with sufentanil at 5 μg. The nerve block procedure was carried out by an experienced anesthesiologist who had participated in this work for more than 2 years and did not engage in the subsequent follow-up procedure. In the case of the interventional group, patients received an ultrasound-guided right serratus anterior plane block (SAPB) and one-puncture of right rectus sheath block (RSB) combined with transversus abdominis plane block (TAPB). In contrast, for the control group, patients received an ultrasound-guided right SAPB and the traditional RSB and TAPB.

For all patients, a high-frequency linear ultrasound probe (Mindray UMT-500) was selected, and a 22G single-use sterile nerve stimulation needle (0.71 × 80 mm, B.Braun Melsungen, Germany) was utilized using the in-plane technique.

#### Serratus anterior plane block

Firstly, the ultrasound-guided right SAPB was carried out. Patients were positioned in the left lateral position, and the probe was placed at the level of the fourth or fifth rib of the right axillary midline (2). After sterilization was completed, the needle was inserted using the in-plane technique. When the needle tip reached the superficial layer of the serratus anterior under direct vision, 2 mL of saline was injected to confirm the position. If no blood or gas was withdrawn, 20 mL of 0.25% ropivacaine was slowly injected, and the local anesthetic spread on the superficial layer of the serratus anterior.

#### One-puncture of RSB combined with TAPB

**Figure 1:**
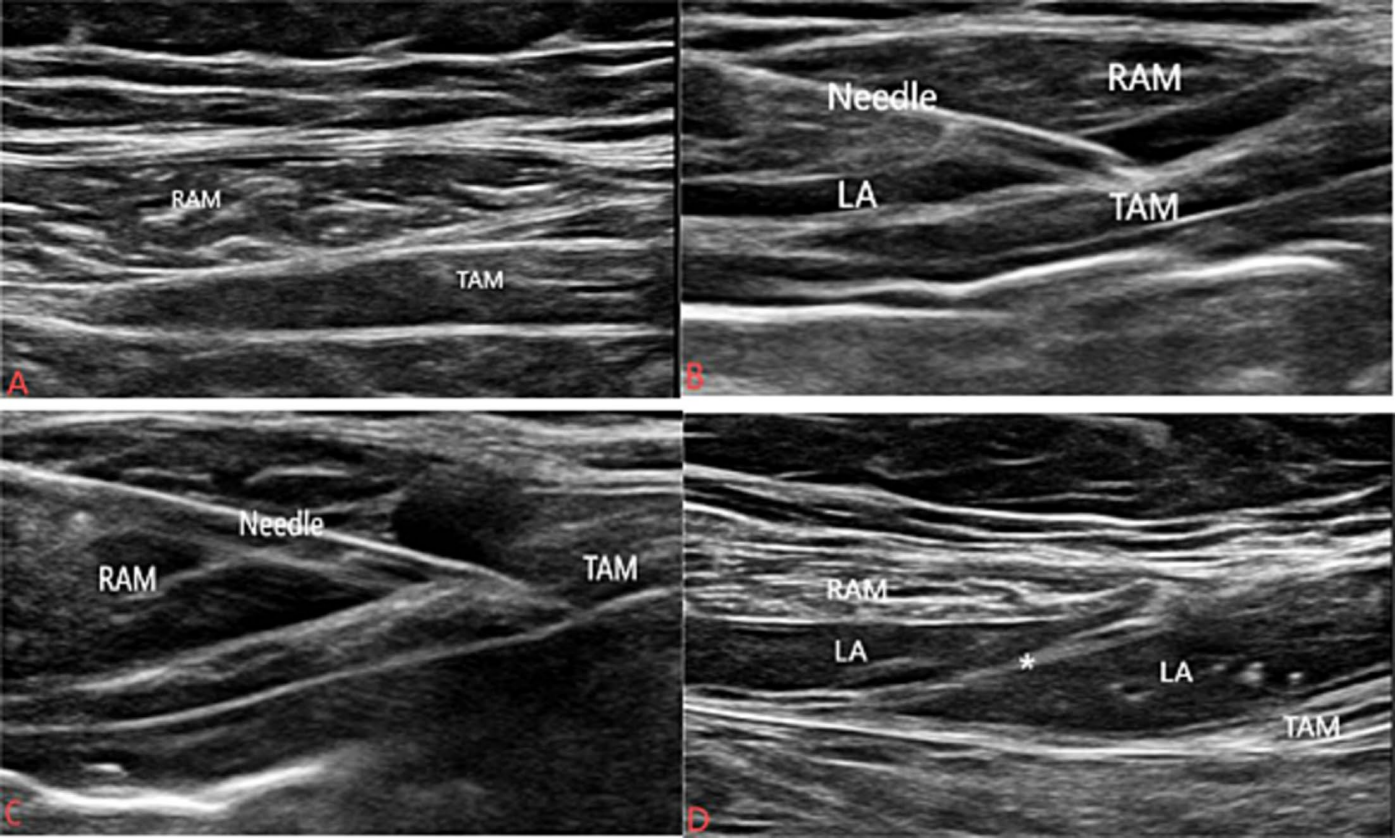
Procedures of one puncture of the RSB and TAPB. **(A)** The ultrasound image of one-puncture of RSB and TAPB. **(B)** The ultrasound images of the RSB by the one-puncture nerve block. **(C)** The ultrasound images of TAPB by the one-puncture nerve block. The needle continues to break through the posterior layer of the rectus abdominis sheath. **(D)** The ultrasound images of the spread of local anesthetic. Asterisk the posterior layer of the rectus abdominis sheath. RAM the rectus abdominal muscle. TAM the transverse abdominis muscle. LA local anesthetic.

Secondly, the ultrasound-guided RSB combined with TAPB was performed. After completing the SAPB, patients were transferred to the supine position. For the one-puncture of RSB combined with TAPB, the probe was placed between the xiphoid and the umbilicus, and then the probe was moved from Hunter’s line toward the midclavicular line along the costal margin, observing the transverse abdominis overlapping the rectus abdominis (18) (Figure 1A). After sterilization, the previous sterile puncture needle was inserted from the inside of the body using the in-plane technique. When the needle tip reached the posterior rectus abdominis sheath and pierced the anterior layer of the posterior sheath, 2 mL of saline was injected to confirm the position. If no blood or gas was withdrawn, 15 mL of 0.25% ropivacaine was slowly injected, and it could be seen that the local anesthetic spread inward of the body (Figure 1B). Then the puncture needle continued to pierce the posterior layer of the posterior sheath, and when the needle tip reached the transversus abdominis plane, the same volume of saline was injected to confirm the position, and again no blood or gas was withdrawn, 15 mL of 0.25% ropivacaine was slowly injected, and it could be seen that the local anesthetic spread along the surface of the transverse abdominis (Figure 1C). One membrane with double capsule could be seen under the ultrasound sign after completing the operation (Figure 1D), and the same procedure was performed on the opposite side.

#### Traditional RSB and TAPB

**Figure 2:**
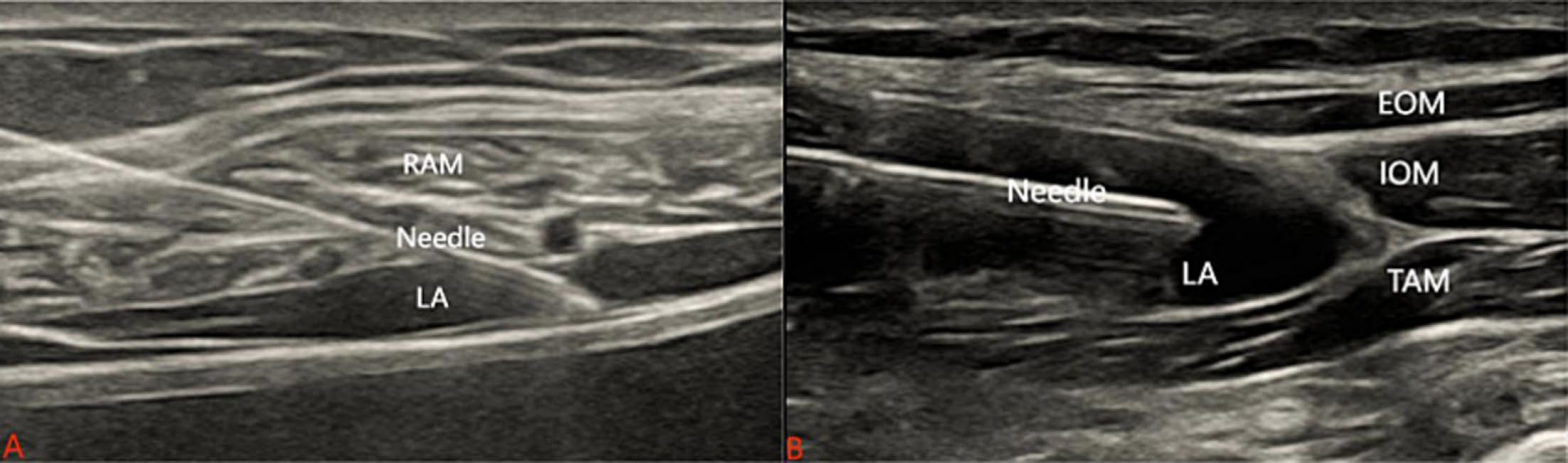
Procedures of the traditional RSB and TAPB. **(A)** The ultrasound images of the traditional RSB. **(B)** The ultrasound images of the traditional TAPB. RAM the rectus abdominal muscle. EOM the external oblique muscle. IOM the internal oblique muscle. TAM the transverse abdominis muscle. LA local anesthetic.

For the traditional RSB and TAPB, traditional RSB was initially conducted. In the supine position, the probe was placed between the xiphoid and the umbilicus, and then by moving the probe, the rectus abdominis muscle and the rectus sheath were found (19). After sterilization, the sterile puncture needle reached the anterior of the posterior rectus sheath using the in-plane technique, 2 mL of saline was injected to confirm the position, if no blood or gas was withdrawn, 15 mL of 0.25% ropivacaine was slowly injected, and it could be seen that the local anesthetic was spreading between the rectus abdominis muscle and the rectus sheath muscle (Figure 2A). Secondly, traditional TAPB was carried out. The subcostal approach was chosen, and the probe was moved to the lateral abdominal wall in the mid-axillary line transversely between the arcus costarum and the iliac crest. The external oblique muscle, internal oblique muscle, and transverse abdominis muscle were visualized under ultrasound. After sterilization, the sterile puncture needle reached the plane of the transversus abdominis muscle using the in-plane technique. 2 mL of saline was injected to confirm the position, if no blood or gas was withdrawn, 15 mL of 0.25% ropivacaine was slowly injected, and the local anesthetic separated the internal oblique muscle and the transversus abdominis muscle (Figure 2B). The procedure was repeated on the opposite side following the same steps.

All nerve blocks were performed under ultrasound guidance with incremental aspiration to minimize intravascular injection risk. Patients remained awake during block administration to enable immediate recognition of local anesthetic systemic toxicity (LAST) symptoms (e.g., perioral numbness, tinnitus), and emergency lipid emulsion and airway management equipment were readily available. 30 min after the operation, the region of the block was assessed by using a 75% ethyl alcohol swab and the region of the disappearing temperature sensation was marked.

#### General anesthesia

Subsequently, all the patients were sent to the surgical room for anesthesia induction after undergoing the previous standard monitoring. Intravenous induction was carried out using 0.02–0.04 mg/kg of midazolam, 0.2–0.4 mg/kg of etomidate, 0.4–0.5 μg/kg of sufentanil, and 0.15–0.20 mg/kg of cisatracurium prior to intubation. After endotracheal intubation, it was ensured that the Bispectral Index (BIS) was maintained between 40 and 60, and that the SpO2, HR, and invasive arterial pressure were within the normal range by inhaling 1–3% of sevoflurane in mixing oxygen and air, pumping 0.05–0.2 μg/kg/min of remifentanil, and intermittently administering intravenous 0.05 mg/kg of cisatracurium. The end-tidal carbon dioxide pressure (PetCO2) was kept between 35 and 45 mmHg with a tidal volume of 6 to 8 mL/kg and a respiratory rate of 10 to 12 times/min. When the HR or the mean arterial pressure (MAP) exceeded 20% from the baseline, 0.1–0.2 μg/kg of sufentanil was administered; if it was below 20% from the baseline, atropine or ephedrine was given. Half an hour before the end of surgery, the patients were administered 0.1 μg/kg of sufentanil and 5 mg of tropisetron.

#### Analgesia methods

All the patients were transferred to the anesthesia recovery room (PACU) after extubation. At the end of the surgery, a patient-controlled intravenous analgesia (PCIA) pump (with a continuous dose of 2.5 mL/h, a bolus dose of 1 mL, and a locking time of 15 min without background infusion) was initiated. It contained 1 μg/mL of sufentanil and 10 mg of tropisetron diluted to 150 mL with normal saline. The postoperative pain was evaluated using the Visual Analog Scale (VAS) (where 0 = no pain and 10 = worst pain) (20). When the VAS was greater than 4 or the pain was unbearable, the PCIA pump was pressed or 5 mg of floxicam was administered immediately as rescue analgesia.

### Follow-up

The anesthesiologist in charge of perioperative data collection and postoperative evaluations was unaware of each patient’s group. The primary outcome was the duration of the nerve block procedure. The secondary outcomes included the convenience of the nerve block operation, the consumption of sufentanil, remifentanil, and cisatracurium, the time of waking up and extubation, the Visual Analog Scale (VAS) scores (for rest, coughing, and deep breathing) at 2, 4, 6, 12, 24 and 48 h after surgery, the Riker SAS at 5, 15 and 30 min, the first time the rescue analgesic was required, the total consumption of sufentanil at 24 and 48 h, the time of the first ambulation, the duration of the postoperative hospital stay, and the incidence of adverse reactions after surgery. The Riker SAS (where 1 = minimal or no response to noxious stimuli; 2 = arousal to physical stimuli but non-communicative; 3 = difficult to arouse but awakens to verbal stimuli or gentle shaking; 4 = calm and follows commands; 5 = anxious or physically agitated but calms on verbal instructions; 6 = requires restraint and frequent verbal reminders of limits; and 7 = attempting to remove the tracheal tube or catheters or striking at staff) (21), and a score of patients between 3 to 5 was considered suitable for the study. The convenience of the nerve block operation was judged by the satisfaction of the operator (where 0 = not at all satisfied, 10 = very satisfied).

### Sample size

The purpose of our study was to test the hypothesis that a single-puncture of RSB and TAPB could shorten the time of nerve block compared to the traditional RSB and TAPB. We recruited 10 patients per group for an initial pilot study and applied the *t*-test, which showed a mean ± standard deviation. The duration of the nerve block procedure was 191.0 ± 13.5 s in the interventional group and 201.3 ± 5.4 s in the control group, with a power of 80% and a significance level of 5%. By using the MedSci Sample Size Tools, we obtained 26 patients (13 in each group), taking into account the dropouts or the possibility of missing data. A minimum of 20 patients for each group was recruited.

### Statistical analysis

Statistical analysis was carried out using SPSS 23.0 (SPSS Inc., Chicago, IL, United States). Continuous data that were normally distributed were presented as the mean ± standard deviation (SD) and analyzed using the t-test, while non-normally distributed data were expressed as medians (interquartile range) and compared using the Kruskal–Wallis H test. Repeated measurement data were analyzed using repeated ANOVA to assess the differences in the interaction effects between groups and time points, followed by the post-hoc Bonferroni correction test to adjust *p* values. Qualitative variables were presented as numbers (%) and analyzed by Pearson’s chi-squared (*χ*^2^) test or Fisher’s exact test. A *p* value <0.05 was considered statistically significant.

## Results

**Figure 3:**
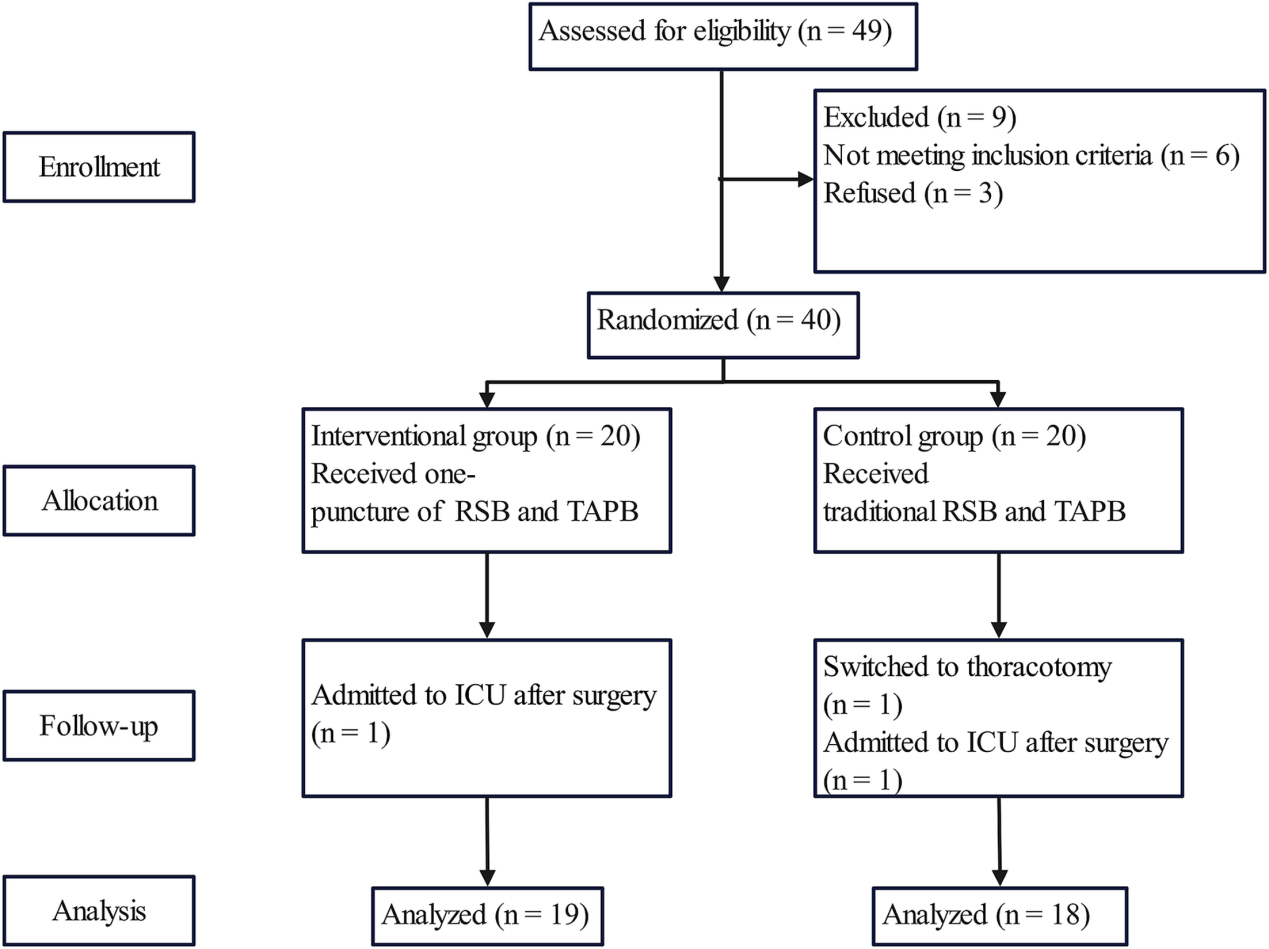
Patients diagram of the study. RSB rectus sheath block, TAPB transverse abdominis plane block.

A total of 49 patients with esophageal cancer were included in our study and randomly divided into the interventional group and the control group. Nine of these patients were excluded, and the remaining 40 patients completed the study, with 20 in the interventional group and 20 in the control group. In the interventional group, 1 patient was admitted to the ICU after surgery. In the control group, 1 patient was switched to thoracotomy while the other patient was admitted to the ICU after surgery. Thirty-seven eligible patients were analyzed (Figure 3). There were no statistically significantly differences in demographic parameters between the two groups (Table 1).

**Table 1:** Demographic data of the patients.

	Control group (*n* = 18)	Interventional group (*n* = 19)	*p* value
Gender			0.653
Male	11 (61.1)	13 (68.4)	
Female	7 (38.9)	6 (31.6)	
Age (years)	67.1 ± 5.6	64.8 ± 7.2	0.279
BMI (kg/m^2^)	24.4 ± 3.1	23.0 ± 1.9	0.111
ASA			0.417
I	5 (27.9)	3 (15.8)	
II	7 (38.9)	8 (42.1)	
III	6 (33.4)	8 (42.1)	

Data are expressed as mean ± standard deviation (SD) or number (%), BMI body mass index, ASA American Society of Anesthesiologists.

The duration of the nerve block procedure in the interventional group was statistically significantly shorter than that in the control group (182.7 ± 13.9 s vs. 199.4 ± 10.9 s, *p* = 0.0003). The convenience of the nerve block operation was statistically significantly lower in the interventional group than that in the control group [9 (8, 10) vs. 8 (7, 8.3), *p* < 0.001; Table 2].

**Table 2:** Time of nerve block and the difficultly of operation skill between the two groups.

	Control group (*n* = 18)	Interventional group (*n* = 19)	*p* value
Duration of nerve block procedure (s)	199.4 ± 10.9	182.7 ± 13.9	0.0003^*^
Convenience of nerve block operation	8 (7, 8.3)	9 (8, 10)	<0.001^*^

Data are expressed as mean ± standard deviation or median (inter quartile range). *p < 0.001, interventional group vs Control group.

**Figure 4:**
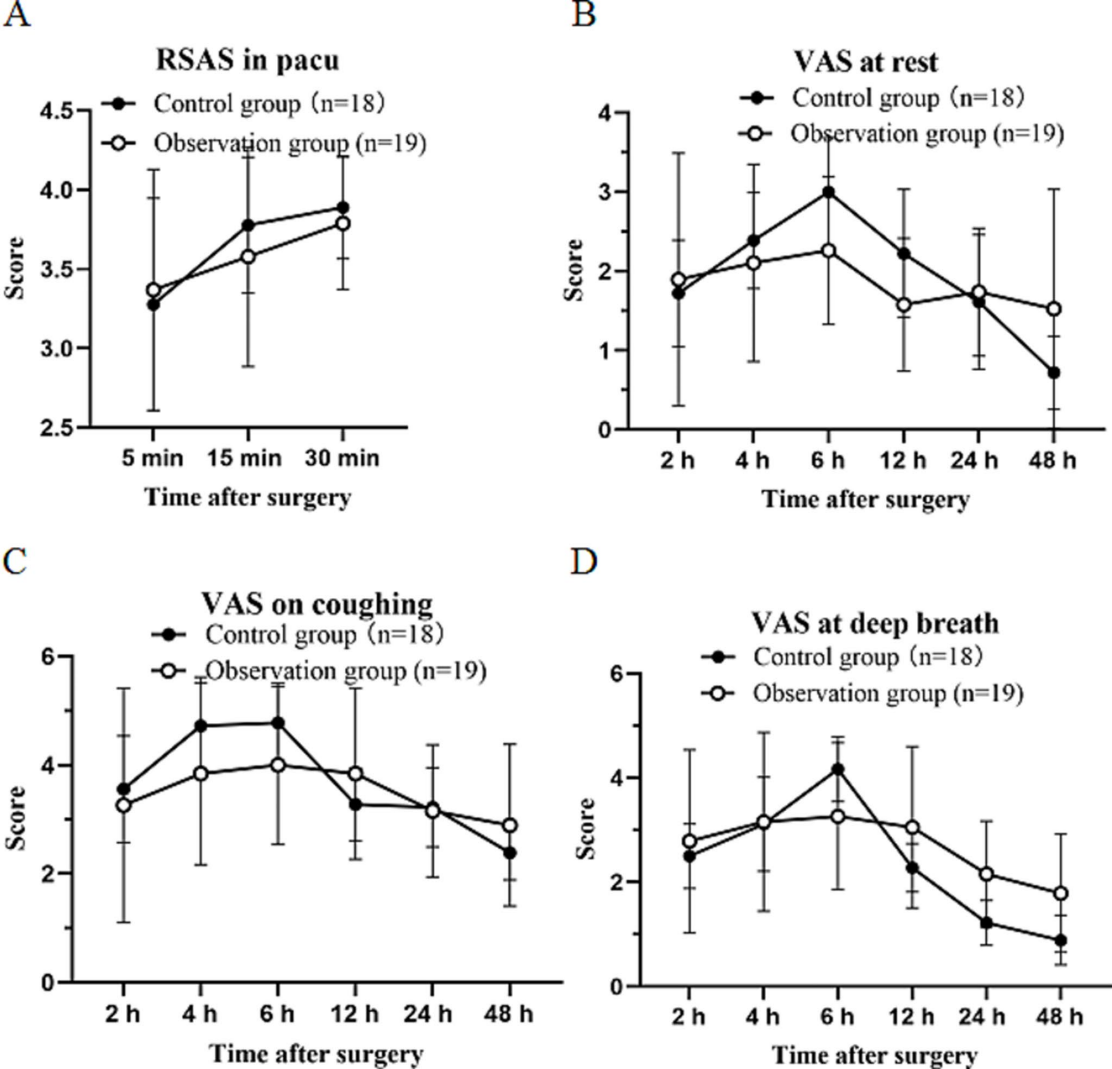
Score of postoperation. **(A)**: RSAS in pacu after surgery. **(B)**: VAS at rest after surgery between the two groups. **(C)**: VAS on coughing after surgery between the two groups. **(D)**: VAS at deep breath after surgery between the two groups. Values are presented as mean ± standard deviation. VAS visual analog scale.

No statistically significant differences were observed in terms of the Riker SAS at 5, 15, and 30 min in the PACU between the two groups, and also the VAS scores (at rest, coughing, and deep breathing) at 2, 6, 12, 24 and 48 h after surgery showed no statistical significant differences between the two groups (*p* > 0.05; Figure 4).

There was no statistical significant differences in terms of the total consumption of sufentanil, remifentanil, and cisatracurium during the operation, the time of the first rescue analgesic requirement, the total amount of analgesia after surgery, the time of the first ambulation, and the duration of the postoperative hospital stay between the two groups (*p* > 0.05; Table 3).

**Table 3:** Perioperative characteristics between the two groups.

	Control group (*n* = 18)	Interventional group (*n* = 19)	*p* value
Sufentanil(ug)	52.8 ± 7.7	55.1 ± 6.4	0.270
Remifentanil (ug)	0.7 ± 0.1	0.6 ± 0.1	0.901
Cisatracurium (mg)	16.1 ± 1.7	15.9 ± 3.0	0.780
Time of surgery (min)	214.9 ± 18.2	222.6 ± 12.2	0.137
Time of anesthesia (min)	199.4 ± 10.8	182.7 ± 13.9	0.052
Time to wake up (min)	25.7 ± 7.1	24.9 ± 12.8	0.822
Time to extubation (min)	28.6 ± 6.2	31.4 ± 6.1	0.499
Volume of bleeding (ml)	97.8 ± 11.3	98.9 ± 10.2	0.758
Volume of urine (ml)	377.8 ± 126.3	418.4 ± 113.6	0.311
Time of first rescue analgesia (min)	221.1 ± 27.8	234.8 ± 31.1	0.167
The use of rescue analgesia, n (%)	5 (27.8)	7 (36.8)	0.648
Time of first PCIA presses (min)	71.8 ± 19.8	68.5 ± 20.9	0.626
Total consumption of sufentanil at 24 h (ug)	61.22 ± 2.0	62.37 ± 3.5	0.235
Total consumption of sufentanil at 48 h (ug)	122.2 ± 3.71	118.1 ± 3.29	0.463
Time of first ambulation (min)	2158.0 ± 42.9	2155.0 ± 52.8	0.867
Hospital stay of postoperative (days)	9.6 ± 1.6	10.7 ± 2.6	0.102

Data are expressed as mean ± standard deviation or number (%), PCIA Patient-controlled intravenous analgesia.

There were no statistical significant differences in terms of adverse reactions such as PONV and dizziness (*p* > 0.05; Table 4).

**Table 4:** Postoperative adverse reactions between the two groups.

	Control group (*n* = 18)	Interventional group (*n* = 19)	*p* value
PONV, *n* (%)	2 (11.1)	1 (5.3)	0.560
Dizziness, *n* (%)	3 (16.7)	2 (10.5)	0.642
Pneumonia, *n* (%)	1 (5.6)	1 (5.3)	>0.999

Data are expressed as number (%), PONV Postoperative nausea and vomiting.

## Discussion

Our study has clearly demonstrated that the implementation of one-puncture of RSB combined with TAPB prior to the induction of general anesthesia in TLE patients can significantly reduce the duration of the nerve block procedure. This not only simplifies the operation but also significantly increases the convenience of the nerve block operation when compared to traditional RSB and TAPB. Moreover, this approach has the potential to provide remarkable postoperative pain relief and play a crucial role in enhancing postoperative recovery after surgery.

China, unfortunately, is a nation with an alarmingly high incidence and mortality rate of esophageal cancer when contrasted with other countries across the globe (1). TLE, on the other hand, represents a completely minimally invasive treatment modality, accompanied by a total of seven incisions, which inevitably cause damage to the normal physical structure of the chest and abdomen. However, these seven incisions are distributed across the upper right chest, abdomen, and left neck, resulting in an extensive surgical range (6). As a consequence, patients often have to endure intense pain following the surgical procedure (22). After the completion of the surgery procedure, patients continue to suffer from severe pain in those incision areas.

There are indeed numerous alternative techniques for pain management, such as epidural anesthesia, thoracic paravertebral nerve block, erector spinal plane block, and local anesthetic drug incisional infiltration (7–9, 23). However, those techniques involve a complex procedure and carries risks such as bleeding, nerve damage, and infection, and in some patients, hypotension and respiratory depression may occur (10, 24). As the needle may pierce the pleura and vessels due to the anatomical structure (9, 25). SAPB has been used to provide excellent postoperative analgesia and reduce the consumption of analgesics after thoracic surgery, the procedure is simple and safe (17, 26, 27). Superficial or deep serratus anterior plane block can provide the same postoperative analgesia in thoracoscopy lobectomy with fewer complications (28, 29). So we selected the superficial serratus anterior plane block in our study. The pain of incisions distribute on abdomen was severe, and acts as a hindrance for patients in coughing and expectorating, particularly, the pain from the abdominal median incision below the xiphoidand significantly affects the exercise of patients’ respiratory functions, ultimately leading to lung atelectasis, pulmonary infections, and other pulmonary complications. This, in turn, prolongs the postoperative recovery period and extends the length of hospitalization time (7). Therefore, there is an imperative need to explore novel methods for abdominal analgesia management in order to improve the recovery of patients and minimize postoperative complications.

For abdominal analgesia, the TAPB has been progressively utilized (30). But the anterior branches of intercostal nerves are arranged in the plane between the internal oblique muscle and the transversus abdominis muscle diagonally, TAPB has shown higher pain scores and greater supplemental morphine requirements compared to intrathecal morphine (31, 32). In actuality, the pathological esophageal tissue is extracted through the median abdominal incision between the xiphoid process and the umbilicus, the patients may still encounter intense pain at the incision sites, and the pain of this incision inhibitis respiration, conventional analgesia techniques are often unable to provide satisfactory analgesia. In our study, we combined with the RSB to compensate for this deficiency. Ultrasound-guided RSB has been demonstrated to enhance pain control and reduce opioid consumption up to 12 h after laparoscopic surgery (33). Previous studies had discovered that the rectus sheath catheters are effective for esophagectomy analgesia, and RSB can provide better analgesia and greater hemodynamic stability for gastrectomy simultaneously (15, 16). RSB offers a more reliable analgesia when compared to thoracic epidural infusion in patients undergoing midline incision laparotomies and does not have associated adverse reactions (26, 34). The incision sites in TLE are an extensive range, so we chose RSB combined with TAPB in our study. The VAS score was signifcantly lower on coughing compared to previous study at 6 h after surgery (2).

The RSB combines with TAPB do indeed reduce the postoperative use of opioids in patients after laparoscopy-assisted surgery (17). However, for the traditonal two-point RSB and TAPB, the duration of the nerve block procedure is lengthy, and the location procedures are complex, which can exacerbate the patient’s discomfort. Moreover, multipoint repeated puncture increases the risk of infection. Hence, we proposed that one-puncture could attain both RSB and TAPB. In fact, when we put US probe at the costal margin one-third outside the rectus muscle, the rectus muscle, the posterior rectus sheath, and the trasversus abdominis muscle below the rectus sheath would be seen in the US image, a layer of membrane separates rectus abdominis and trasversus abdominis muscle (35). Our previous study had found that one-puncture could arrive the anterior layer of the posterior rectus sheath and transversus abdominis muscle plane in laparoscopic upper abdominal surgery (18). Nevertheless, the one-puncture of RSB combined with TAPB method has not yet been investigated in achieving pain relief in TLE. Therefore, in this prospective randomized controlled trial, we compared the feasibility and efficacy of one-puncture of RSB combined with TAPB (interventional group) with the traditional RSB and TAPB (control group) in terms of the analgesic effect and recovery after TLE.

In our study, the duration of the nerve block procedure was 182.7 ± 13.9 s in the interventional group and 199.4 ± 10.9 s in the control group, with a statistically significantly shorter time in the interventional group. The convenience of the nerve block operation was statistically significantly higher in the interventional group at 9 (8, 10) than that in the control group at 8 (7, 8.3). For nerve block, we typically choose the sterile nerve stimulation needle, and the point of the needle will become blunt after puncturing more than twice for one patient. The traditional nerve block requires repeating five operations, which increases the difficulty of the operation skill and the time for nerve block, and repeated operations also raise the risk of infection. For the one-puncture nerve block, we only need to repeat it three times, which increases the convenience of the nerve block operation. At the same time, it reduces the patients’ discomfort and the incidence of infection (2).

In our novel study, we found no statistically significant difference in terms of RSAS in the PACU and VAS score at different points after surgery. The VAS score was the highest at 6 h after surgery in both groups, which might be due to the complete metabolism of the anesthetic drug at this time point. These findings were consistent with previous studies which confirmed that the multipoint fascia plane block technique could reduce postoperative pain in TLE (2). The rescue of analgesia and the total use of sufentanil showed no statistically significant differences between the interventional group and the control group. This result indicates that the one-puncture of RSB combined with TAPB has the same effect on pain relief as the traditional RSB and TAPB in TLE.

The local anesthetic was 0.25% ropivacaine without epinephrine for both groups, and this concentration was safe and also effective in relieving postoperative pain (36). We carried out the nerve block when the patients were awake so that we could observe the toxic reaction of the local anesthetics, while the total ropivacaine dose approached the upper safety limit, strict adherence to ultrasound-guided techniques and real-time monitoring mitigated systemic toxicity risks, and there was no toxic reaction in interventional group and control group. Before applying general anesthesia, we tested the range of the block 30 min after conducting the nerve block, and the region of temperature sensation disappeared from T6 to L1, which was in line with the previous study (33).

Several parameters were observed, including the dosage of anesthesia, the time of waking up and extubation during the surgical procedure. These parameters showed no statistically significant differences between the interventional group and the control group. During the operation, the dosage of sufentanil in the interventional group and the control group was 55.1 ± 10.4 and 52.8 ± 7.7 respectively, with no statistically significant differences in these two groups, which may indicate that multiple regional anesthestics can provide satisfactory analgesia during the operation. The time to extubation was approximately 30 min, general anesthesia combined with multiple regional anesthestics can reduce the use of anesthetics, especially opioids, and accelerate the patient’s recovery. At the same time, these two groups had a very low incidence of adverse reactions including PONV, dizziness, and pneumonia, with no statistically significant differences. This might be due to two reasons: (1) The multiple regional anesthestics provide postoperative analgesia, so the patients can cough and expectorate effectively, and thus the incidence of pneumonia is low. (2) Multiple regional anesthestics can reduce the use of opioids which is considered the main reason for PONV and dizziness.

There exist several limitations in this study. Firstly, the study was conducted in a single-center and with a relatively small sample size, which restricts the generalizability of the findings. Hence, multicenter studies are required to assess the feasibility and efficacy of multiple regional anesthestics. Secondly, we completed the nerve block prior to general anesthesia and injected a total of 80 mL of 0.25% ropivacaine, and this volume might potentially cause a toxic reaction. The 0.25% ropivacaine is utilized in the majority of trials, and the patients were awake, enabling us to observe the toxic reaction of the local anesthetics. At the same time, we carried out the nerve block under ultrasonic guidance to avoid the needle damaging blood vessels. Thirdly, the total ropivacaine dose (200 mg) approached the upper safety limit for non-obstetric use (37) and the fixed-dose regimen did not adjust for individual body weight. Although no systemic toxicity was observed in our study, this represents a critical limitation. Future trials should prioritize weight-based dosing (e.g., 3 mg/kg) to ensure compliance with safety guidelines across all body habitus types.

## Conclusion

This single-center, prospective, and randomized study demonstrated that the one-puncture of RSB in combination with TAPB can offer sufficient analgesia for patients undergoing TLE, and it can significantly reduce the duration of the nerve block procedure and enhance the convenience of the nerve block operation with few adverse reactions. The results of this research can accelerate the postoperative recovery and provide a more feasible and effective perioperative analgesia strategy in TLE.

## Data Availability

The raw data supporting the conclusions of this article will be made available by the authors, without undue reservation.
