# Supplementary material for: The feasibility and effectiveness of one-puncture of rectus sheath block combined with transverse abdominis plane block in patients undergoing thoracoscopic-laparoscopic radical esophagectomy: a prospective randomized controlled study
Source: Front Med (Lausanne). 2025 Apr 7;12:1568464. doi: 10.3389/fmed.2025.1568464 (PMC12009887; doi:10.3389/fmed.2025.1568464)
Supplement: Supplementary file 1 [file Data_Sheet_1.pdf]

There were no statistically significant differences in terms of HR and MAP between the two groups at the baseline, after the nerve block, before the thoracoscopic incision, after the thoracoscopic incision, before the laparoscopic incision, and after the laparoscopic incision ($P > 0.05$) (Fig).
