# Supplementary figures and images for: The feasibility and effectiveness of one-puncture of rectus sheath block combined with transverse abdominis plane block in patients undergoing thoracoscopic-laparoscopic radical esophagectomy: a prospective randomized controlled study
Source: Front Med (Lausanne). 2025 Apr 7;12:1568464. doi: 10.3389/fmed.2025.1568464 (PMC12009887; doi:10.3389/fmed.2025.1568464)

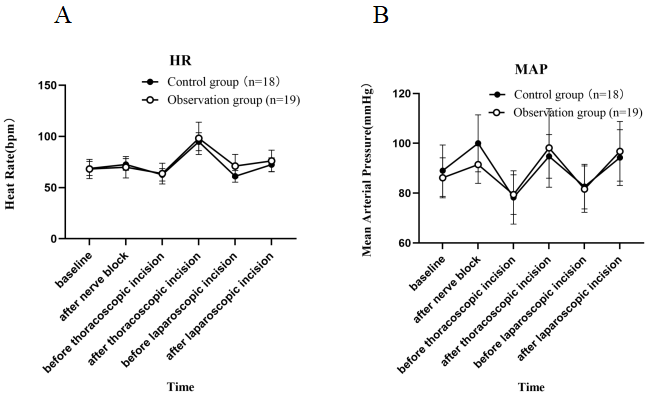

Supplement: Supplementary file 2 [file Image_1.tif]
